# Extreme Intrahepatic Cholestasis of Pregnancy With Fetal Ascites and Neonatal Meconium Peritonitis

**DOI:** 10.7759/cureus.102636

**Published:** 2026-01-30

**Authors:** Wojciech J Bajda, Bronisława Pietrzak, Julia Sosin, Bożena Kociszewska-Najman, Maria Wilińska, Irena Wysocka, Aleksandra Jasińska, Michał Lipa, Dorota Bomba-Opoń, Mirosław Wielgoś

**Affiliations:** 1 Department of English, Medical University of Warsaw, Warsaw, POL; 2 Department of Obstetrics and Perinatology, National Medical Institute of the Ministry of Interior Affairs and Administration, Warsaw, POL; 3 Department of Neonatology and Rare Diseases, Medical University of Warsaw, Warsaw, POL; 4 Department of Neonatal Physiology, Pathology and Intensive Care Diseases, National Medical Institute of the Ministry of Interior Affairs and Administration, Warsaw, POL; 5 Department of Pediatric Surgery, Pediatric Urology and Pediatrics, Medical University of Warsaw, Warsaw, POL; 6 Department of Obstetrics and Perinatology, National Medical Institute of the Ministry of Internal Affairs and Administration, Warsaw, POL; 7 Department of Obstetrics and Gynecology, Collegium Medicum of Jan Kochanowski University, Kielce, POL

**Keywords:** fetal ascites, intrahepatic cholestasis of pregnancy, meconium peritonitis, pediatric ileostomy, severe maternal hypercholanemia

## Abstract

Intrahepatic cholestasis of pregnancy (ICP) is a rare but potentially severe condition with significant maternal and fetal risks. A 28-year-old gravida 2 para 1 woman presented at 21 weeks’ gestation with pruritus and jaundice and was diagnosed with severe ICP (bile acids 297 μmol/L). Despite ursodeoxycholic acid (UDCA) treatment, escalation with rifampicin and antihistamines was required. At 23 weeks, fetal ultrasound revealed massive ascites and features suggestive of meconium peritonitis (MP). Intensive maternal-fetal monitoring allowed pregnancy prolongation to 36 weeks, when cesarean delivery was performed. The neonate, initially stable, developed bowel obstruction on day 7 and underwent surgical resection with ileostomy, followed by gradual recovery. This case illustrates the potential for rare but severe fetal and neonatal complications in the setting of extreme ICP. Early recognition, multidisciplinary management, and individualized delivery planning are crucial for optimizing maternal and neonatal outcomes.

## Introduction

Intrahepatic cholestasis of pregnancy (ICP) is the most common gestational liver disease, typically presenting in the third trimester, with an estimated occurrence between 0.02% and 2.4% of all pregnancies globally [1]. While maternal symptoms are generally benign, ICP poses much greater risks to the fetus. These include an increased risk of preterm birth, meconium-stained amniotic fluid (MSAF), fetal bradycardia, intrauterine asphyxia, and, in severe cases, intrauterine fetal death [2].

ICP is diagnosed primarily based on maternal total bile acids (TBA) exceeding 10 μmol/L, with elevated alanine aminotransferase (ALT) serving as a supportive finding, after exclusion of other causes of liver dysfunction. The risk of fetal complications increases with TBA concentrations, especially with severe ICP, classified as TBA equal to or exceeding 100 μmol/L [3]. Meconium peritonitis (MP) is a rare inflammatory condition caused by fetal bowel perforation, with subsequent leakage of meconium into the peritoneal cavity, and its occurrence in the setting of ICP is exceptionally uncommon. This typically presents as fetal ascites and can be accompanied by intraperitoneal calcifications [4]. We report a case of very early-onset, extreme ICP with a peak TBA of 297 μmol/L, complicated by massive fetal ascites and neonatal MP requiring surgical treatment. We also discuss the implications of this presentation for perinatal management.

## Case presentation

A 28-year-old gravida 2 para 1 woman at 21+1 weeks’ gestation was referred by her managing physician and admitted to the Department of Obstetrics and Perinatology with suspected ICP. She reported seven days of pruritus (on the legs, forearms, and hands) and new-onset jaundice. She had no significant medical history. Laboratory tests showed markedly elevated liver parameters (Table 1). Viral, parasitic, and genetic causes were excluded; serologies for hepatitis A, B, C, D, E, cytomegalovirus, Epstein-Barr virus, human immunodeficiency virus, parvovirus B19, and *Toxoplasma gondii* were negative, and amniocentesis revealed no abnormalities. Maternal ultrasound demonstrated a thin-walled gallbladder containing multiple stones. Fetal ultrasound showed free intraperitoneal fluid.

**Table 1:** Laboratory findings of a patient with intrahepatic cholestasis of pregnancy across different gestational ages ALT: alanine transaminase; AST: aspartate transaminase; TBA: total bile acids

Parameters	Reference Range in Pregnancy	21+1 Weeks	22 Weeks	22+2 Weeks	36+2 Weeks
ALT (U/L)	≤ 35	382	179	130	57
AST (U/L)	≤ 35	242	60	53	71
Bilirubin (mg/dL)	0.1-1.0	3.45	3.53	2.99 (Direct 2.90; Indirect 0.09)	1.42
TBA (μmol/L)	< 10	297	237	117	114

Based on these findings, treatment with ursodeoxycholic acid (UDCA) 900 mg/day and clemastine was initiated. Due to worsening of pruritus after two days, UDCA was increased to 1,200 mg/day (600 mg morning, 300 mg afternoon, 300 mg evening). Rifampicin 600 mg/day was added for refractory cholestasis, along with cetirizine 10 mg in the morning and hydroxyzine 10 mg at night. Pruritus improved over the next three days.

**Figure 1:**
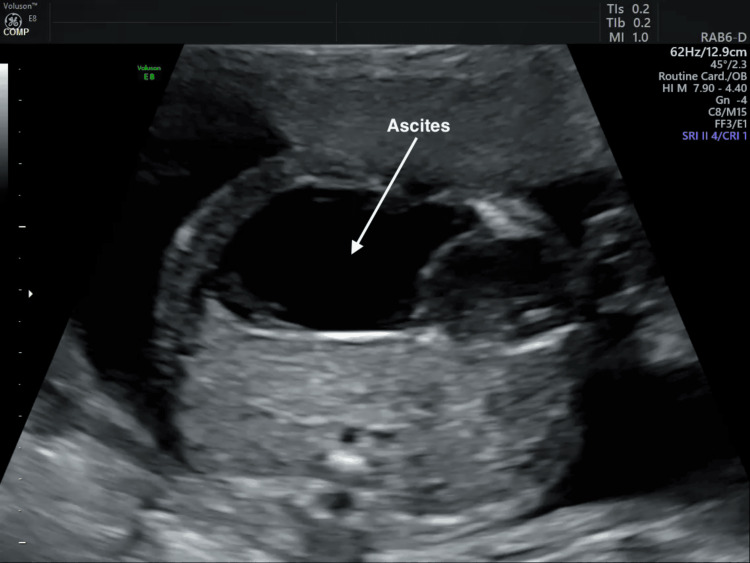
Prenatal ultrasound showing massive fetal ascites Transabdominal ultrasound at 23 weeks' gestation showing a markedly distended fetal abdomen with free intra-abdominal fluid consistent with massive fetal ascites.

At 22 weeks, follow-up laboratory testing and hepatology consultation demonstrated a marked decline in transaminases (ALT from 382 to 179 U/L and aspartate transaminase (AST) from 242 to 60 U/L) and a substantial reduction in TBAs (from 297 to 237 μmol/L), with stable bilirubin levels (Table 1). By 22+2 weeks, TBAs had further decreased to 117 μmol/L, and bilirubin fractions (mg/dL) were assessed to clarify etiology and exclude hemolysis. Serial fetal ultrasounds demonstrated increasing intra-abdominal fluid and relative cardiomegaly. At 23 weeks, ultrasound revealed a fluid collection measuring 40 × 21 × 57 mm and a hyperechogenic, avascular mass (20 × 19 × 21 mm) consistent with suspected MP (Figure 1). Maternal ultrasound showed an enlarged gallbladder with numerous stones (80 × 37 mm).

At 23+2 weeks, magnetic resonance cholangiopancreatography confirmed multiple gallstones without cholecystitis or biliary duct dilation. Surgical consultation found no indication for cholecystectomy. Repeat fetal ultrasound was unchanged. At 23+5 weeks, oral vitamin K (10 mg/day) was started due to impaired absorption from cholestasis and associated bleeding risk. Subsequent fetal ultrasounds continued to suggest MP, though findings were regressing. The patient was discharged at 24 weeks in stable condition with recommendations for close follow-up.

At 36+1 weeks, the patient was readmitted for persistent severe ICP, asymptomatic cholelithiasis, and ongoing suspicion of MP. Due to persistent severe ICP, labor induction with a Foley catheter and oxytocin was attempted but failed; therefore, cesarean delivery was performed at 36+3 weeks without complications. A late-preterm male infant was delivered in good condition (birth weight 2,800 g (48^th^ percentile), length 49 cm (71^st^ percentile), head circumference 34 cm (76^th^ percentile)) with Apgar scores of 8/8/9/9 [5]. Expanded screening ruled out cystic fibrosis. The infant passed meconium shortly after birth, and arterial blood gases were within the expected limits for a late preterm newborn. The mother’s ICP symptoms resolved within three days postpartum. Neonatal abdominal X-ray and ultrasound were normal, and feeding was initiated with good tolerance.

On day seven, the infant developed irritability, grunting, feeding refusal, and abdominal distention. Vitals remained stable, but abdominal X-ray showed signs of intestinal obstruction with dilated bowel loops and fluid-gas levels. Enteral feeding was stopped, and an emergency laparotomy was performed. Intraoperatively, there were extensive interloop adhesions; the terminal ileum and cecum were thickened, ischemic, and adherent. Surgical management included resection, closure of the colon, and creation of an end ileostomy. Peritoneal fluid culture grew *Escherichia coli* sensitive to piperacillin-tazobactam, which was administered for 14 days.

Postoperatively, the infant required mechanical ventilation and was extubated to high-flow nasal cannula therapy on day two. Trophic maternal milk feeds were initiated on day three. Despite the mother following a cow-milk protein-free diet, stools remained watery, with significant losses via the stoma. Prompting transition to an amino acid-based elemental formula, with good tolerance.

The infant was discharged on day 38 in good condition, tolerating full elemental formula feeds, with a functioning stoma. At discharge, weight was 3,330 g (11^th^ percentile), head circumference 37.5 cm (88^th^ percentile), and length 56 cm (94^th^ percentile). At six months, surgical stoma closure was successfully performed. At 11 months, the child showed normal growth and neurodevelopment, weighed 11 kg, was breastfed, and consumed a diversified diet.

## Discussion

This case highlights a rare and severe form of early-onset ICP, characterized by exceptionally high maternal bile acid levels and complicated by massive fetal ascites and MP requiring neonatal surgical intervention. Although ICP is the most common hepatic disorder of pregnancy and is associated with stillbirth, preterm delivery, and MSAF, the development of fetal bowel perforation and MP in this context is extremely uncommon [6].

ICP typically presents in the third trimester and is associated with risk factors such as personal or family history of biliary disease, hepatitis C infection, twin pregnancy, in vitro fertilization, and advanced maternal age [2]. Recurrence is high, occurring in 40% to 60% of subsequent pregnancies [6]. In contrast, MP is rare (one in 30,000), and over half of affected neonates require surgery, with recent survival rates ranging from 80 to 92.3% [7]. In our patient, the only identifiable ICP risk factor was cholelithiasis. The early onset and rare coexistence of MP make this case especially distinctive.

The pathogenesis of ICP is multifactorial, involving hormonal, genetic, and environmental influences. Elevated estrogen levels, often seen in multiple gestations, are associated with increased ICP risk [6]. In contrast, MP is generally attributed to congenital intestinal obstruction, wall dysplasia, or, less commonly, to cystic fibrosis or chromosomal anomalies, though some cases remain idiopathic [7]. 

UDCA remains the first-line treatment for ICP at 10-20 mg/kg/day, with rifampicin recommended for refractory disease to enhance bile acid elimination [6]. Antihistamines and hydroxyzine can provide symptomatic relief, and dexamethasone may be administered when early delivery is anticipated to support fetal lung maturity. Vitamin K supplementation is advised due to fat-soluble vitamin malabsorption and increased risk of hemorrhage [6]. In this case, persistently elevated bile acids despite standard UDCA therapy indicated refractory ICP, prompting the addition of rifampicin in accordance with current recommendations. Close maternal-fetal surveillance was crucial to safely prolonging the pregnancy to 36 weeks, with ultrasound and cardiotocography performed every two weeks until 30 weeks and weekly thereafter. Management of MP is primarily surgical after birth, typically requiring partial bowel resection and stoma formation, as seen in our patient [8].

The relationship between ICP and MP remains poorly understood and rarely reported. ICP is known to increase the incidence of MSAF to 16% to 58% and up to 100% in cases of intrauterine death, with the risk rising at higher bile acid concentrations [9]. Studies suggest that elevated bile acids stimulate fetal colonic motility, causing premature meconium passage [10], and contribute to fetal distress and hypoxia through placental vasoconstriction and induction of abnormal fetal heart rhythms [11-12]. In this case, however, the amniotic fluid remained clear, and the neonatal condition was favorable.

Despite these associations, MP remains exceptionally rare. If ICP commonly causes fetal bowel perforation, the incidence of MP would be far higher given the global prevalence of ICP. Current epidemiological data therefore suggest that MP usually arises from distinct structural or ischemic mechanisms rather than maternal metabolic disease. However, in cases of severe ICP, extreme maternal hypercholanemia may act as a modifying factor rather than a direct cause. Very high bile acid levels can affect the fetoplacental circulation and fetal heart function, which may make an already abnormal fetal bowel more vulnerable to injury [6]. In this context, the rare coexistence of ICP and MP may reflect the co-occurrence of severe maternal cholestasis with underlying fetal bowel disease, as described in MP [7]. Emerging evidence further supports this interpretation, including a recent 10-year retrospective case-control study by Feng et al., which suggests a dose-dependent increase in risk: maternal TBA levels of 10-39 μmol/L increased MP risk fivefold. In comparison, levels ≥40 μmol/L increased risk up to tenfold [13]. 

Our patient’s peak TBA level of 297 μmol/L far exceeded the threshold for severe ICP and is among the highest reported in the current literature. The presence of massive fetal ascites (Figure 1) and subsequent diagnosis of MP support the possibility that, in rare cases, extreme maternal hypercholanemia may contribute to fetal bowel injury. Close and frequent monitoring is therefore essential in severe ICP, particularly at markedly elevated TBA levels. Following guideline recommendations that patients with TBA ≥100 μmol/L should be offered delivery at 36 0/7 weeks due to substantially increased risk of stillbirth [14], our patient, whose TBA rose to 114 μmol/L at 36+2 weeks (Table 1), was managed accordingly. Although severe ICP was the primary indication for induction of labor, cesarean delivery was ultimately performed following failed induction, with persistent fetal ascites and ongoing suspicion of MP further supporting the decision.

## Conclusions

This case underscores the importance of heightened vigilance and rigorous fetal monitoring in pregnancies complicated by severe ICP, especially when maternal bile acid levels are markedly elevated. Although the association between ICP and fetal MP is rare, clinicians should maintain a high level of diagnostic awareness in cases of early-onset or severe ICP and remain attentive to atypical fetal findings, such as massive ascites. Early multidisciplinary involvement and individualized management, with readiness to escalate care, including timely delivery and neonatal surgical intervention, are crucial for optimizing maternal and neonatal outcomes. Our experience suggests that, in rare cases, extreme maternal hypercholanemia may contribute to fetal bowel injury. Prompt recognition and tailored perinatal management can minimize morbidity and support favorable outcomes, even in the most complex presentations.
